# Recent Insight on the Non-coding RNAs in Mesenchymal Stem Cell-Derived Exosomes: Regulatory and Therapeutic Role in Regenerative Medicine and Tissue Engineering

**DOI:** 10.3389/fcvm.2021.737512

**Published:** 2021-10-01

**Authors:** Tarun Pant, Matea Juric, Zeljko J. Bosnjak, Anuradha Dhanasekaran

**Affiliations:** ^1^Department of Medicine, Medical College of Wisconsin, Milwaukee, WI, United States; ^2^Department of Biophysics, Medical College of Wisconsin, Milwaukee, WI, United States; ^3^Department of Physiology, Medical College of Wisconsin, Milwaukee, WI, United States; ^4^Centre for Biotechnology, Anna University, Chennai, India

**Keywords:** MSCs, non-coding RNAs, regenerative medicine, tissue engineering, exosomes

## Abstract

Advances in the field of regenerative medicine and tissue engineering over the past few decades have paved the path for cell-free therapy. Numerous stem cell types, including mesenchymal stem cells (MSCs), have been reported to impart therapeutic effects *via* paracrine secretion of exosomes. The underlying factors and the associated mechanisms contributing to these MSC-derived exosomes' protective effects are, however, poorly understood, limiting their application in the clinic. The exosomes exhibit a diversified repertoire of functional non-coding RNAs (ncRNAs) and have the potential to transfer these biologically active transcripts to the recipient cells, where they are found to modulate a diverse array of functions. Altered expression of the ncRNAs in the exosomes has been linked with the regenerative potential and development of various diseases, including cardiac, neurological, skeletal, and cancer. Also, modulating the expression of ncRNAs in these exosomes has been found to improve their therapeutic impact. Moreover, many of these ncRNAs are expressed explicitly in the MSC-derived exosomes, making them ideal candidates for regenerative medicine, including tissue engineering research. In this review, we detail the recent advances in regenerative medicine and summarize the evidence supporting the altered expression of the ncRNA repertoire specific to MSCs under different degenerative diseases. We also discuss the therapeutic role of these ncRNA for the prevention of these various degenerative diseases and their future in translational medicine.

## Introduction

Over the past several decades, incidences of damage, failure, dysfunction of multiple tissues and organs have reached epidemic proportions (1–3). However, scarcity of donors, limited availability of autologous tissues, risk of chronic tissue rejection, lack of a cost-effective therapeutic strategy, and the overall financial burden of caring for affected individuals present challenges in developing advanced therapeutic options (4–9).

The advent of regenerative medicine has provided immense hope to the primary researcher and clinician to use stem cells with a determined aim of rejuvenating the damaged tissues and organs (10–12). Numerous animal models have been used to test stem cell types (e.g., embryonic, induced, and adult) over the past years for autologous and allogeneic therapies (13–16). Although having a relatively limited differentiation potential in comparison to the embryonic stem cells (ESCs) and induced pluripotent stem cells (iPSCs), the combination of factors like autologous cell therapy, reduced possibility of teratoma formation, immunological intolerance, xenogeneic contaminations, lack of ethical concern, and the reduced financial burden have made mesenchymal stem cells (MSCs), an ideal choice for the regenerative cell therapy (17–21). Despite the promising therapeutic potential of MSCs in regenerative medicine, few of the critical questions, such as what are the precise molecular mechanisms responsible for providing the host's immune tolerance against MSCs, is the clinical application of MSCs safe, and how can we increase the scalability and yield per batch, remain unanswered and have imposed significant challenges in their clinical translation, prompting researchers to explore alternative tissue regeneration approaches.

Recent studies propose that MSCs profoundly manifest their action by the paracrine secretion of extracellular vesicles (EVs), particularly exosomes (22–25). The unique potential of these MSCs-derived exosomes (MSCs-Exos) to recapitulate the stem cell properties have paved the path for a “cell-free” therapy in the field of regenerative medicine. Furthermore, in recent years, it has been substantially demonstrated that MSCs-Exos cargo is enriched in distinct ncRNAs, specially-microRNAs (miRNAs), long non-coding RNAs (lncRNAs), circular RNAs (circRNAs), and Piwi-interacting RNA (piRNAs), mediating tissue communication, modifying cellular phenotype, regulating wide range of biological, pathophysiological processes making them a relevant drug delivery and therapeutic option in the field of regenerative medicine (26–30).

In the current review article, we aim to summarize the emerging role of MSCs-Exos-derived ncRNAs (MSCs-Exos ncRNAs) in regenerative medicine and diseases. We further emphasize the regulatory mechanisms, therapeutic approaches, ongoing clinical applications, and the challenges associated with MSCs-Exos ncRNAs in the field of regenerative medicine and tissue engineering.

## MSC-Exo's Biogenesis

**Figure 1:**
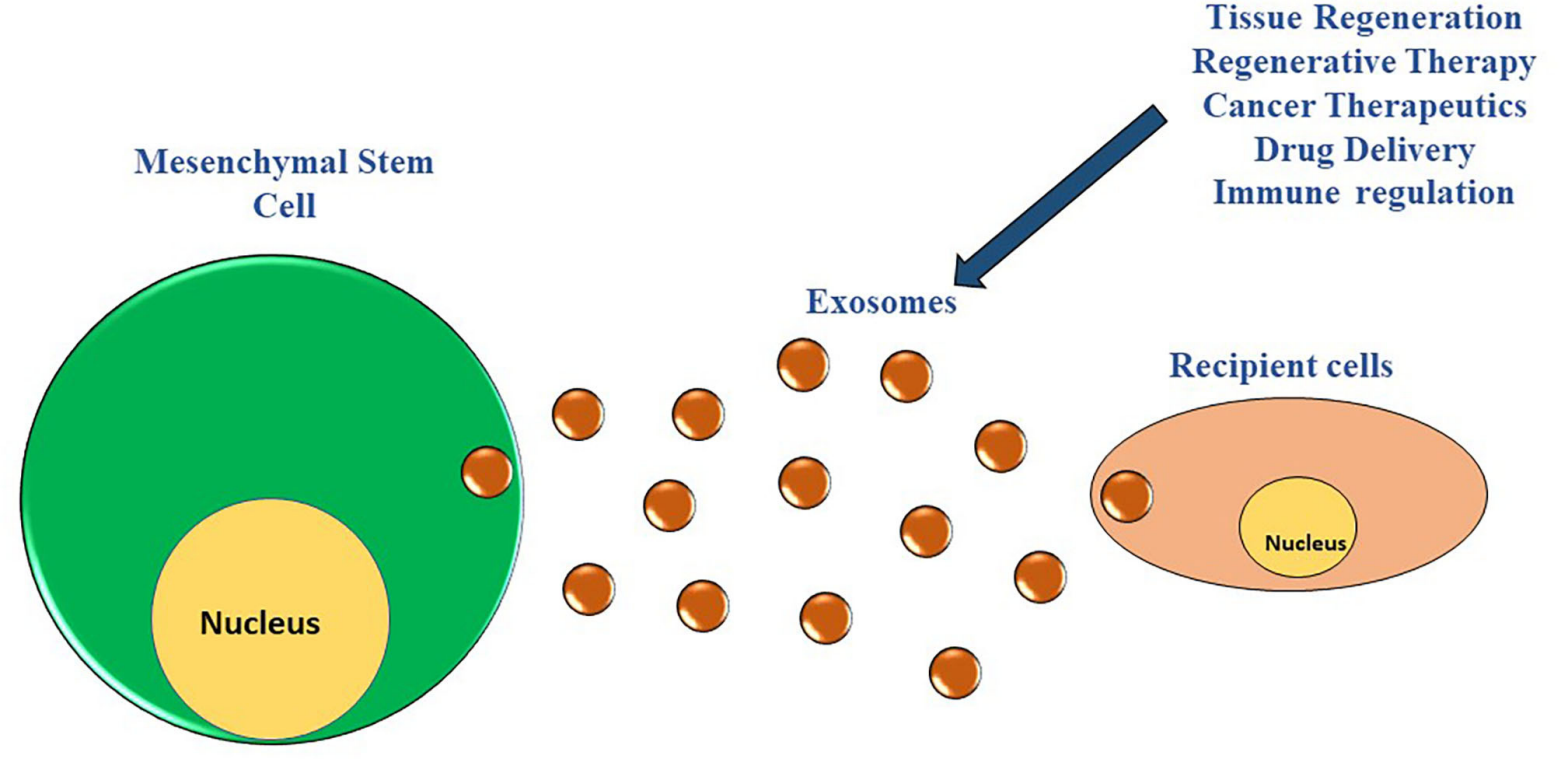
MSCs derived exosomes and their interaction with the neighboring cells. The MSCs have the potential to modulate numerous biological processes of numerous cell types in their vicinity *via* paracrine secretion of exosomes.

MSCs are stromal, nonhematopoietic cells generally obtained from different sources including bone marrow, adipose tissue, umbilical cord, placenta, amniotic fluid, and dental pulp (31–37). The characterization of the MSCs depends upon the expression of specific cell surface markers (CD29, CD37, CD73, CD90, CD102, CD105, and CD166) and their potential to differentiate into multiple cell types such as osteocytes, chondrocytes, and adipocytes under appropriate, stimulating culture conditions (38–43). Recent clinical and preclinical trials have demonstrated the potential of MSCs to be an excellent autologous cell source treating numerous diseases including diabetes, myocardial infarction, osteoarthritis, Alzheimer's, both in animal models and patients (44–49). Although MSCs-based therapy has proven beneficial in treating a wide range of diseases, we still have incomplete knowledge regarding their mechanism of action. Emerging studies in recent years have found that MSCs mediate their action *via* the paracrine secretion of exosomes (Figure 1), regulating numerous biological processes (50).

Exosomes are membrane-enclosed spherical or cup shaped endocytic vesicles with a size ranging from 40 to 150 nm formed intracellularly in the cellular multivesicular bodies and released from numerous cell types (51–53).

Exosome biogenesis occurs *via* three pathways: ESCRT-dependent, ESCRT independent, or direct budding of the plasma membrane. In the first two pathways, an early endosome is formed by the inward budding of the endosomal membrane to generate intraluminal vesicles (ILVs). These ILVs accumulate to form multivesicular bodies (MVBs), which fuse with the plasma membrane (facilitated by Rab GTPases). The ILVs are then released from cells as exosomes, where they are taken up by recipient cells *via* endocytosis, direct binding, or ligand-receptor binding. The Endosomal Sorting Complexes Required for Transport (ESCRT) includes four distinct proteins: ESCRT 0, I, II, and III. Briefly, ESCRT 0 relegates ubiquitinated proteins within the endosomal membrane and recruits ESCRT I and II. ESCRT I and II bind near ubiquitinated proteins on the outer surface of the endosomal membrane, “tagging” them for recruitment within newly forming intraluminal vesicles in the MVB. ESCRT III then sequesters MVB proteins, finalizing the process of exosome formation. Exosome biogenesis *via* the ESCRT-independent pathway was discovered recently after knock-out studies involving the ESCRT complex proteins showed that the cells could continue the process of exosome formation and release. This pathway involves lipids such as sphingolipids and ceramides and proteins such as heat shock proteins and tetraspanins (54–56). Why these distinct pathways exist, and which pathway is used in cells is still poorly understood. Investigators have used different approaches to increase the angiogenic potential of exosomes released by the stem cells (57). The release of exosome can be considerably increased *in vitro* using stress conditions that mimic organ injuries, such as hypoxia, irradiation, or drug treatments.

The MSC-Exos have been tested widely on human patients of ischemic heart injury, cerebrovascular disease, and liver fibrosis as an alternative to MSCs themselves (58–61). Intriguingly, numerous advantages over MSCs such as increased viability, higher uptake, lower immune response, reduced risk of embolism, and potential to cross the blood-brain barrier have made MSC-Exos a promising candidate emerging as an effective “cell-free” therapeutic approach in the field of regenerative medicine (22, 62–64).

In recent years, considerable amounts of research depict MSCs-Exos ability to facilitate the exchange of genetic and epigenetic information intercellularly leading to the modulation of neighboring and distant cells' gene expression in both healthy and disease states (65). Preclinical data further demonstrate that the acquired repertoire of biological cargo, primarily ncRNAs of MSC-Exos, has been found to effectively disseminate biological information between cells and regulate the inflammatory apoptotic pathways in various and disease states (64, 66).

In the next section, we represent the overview of ncRNAs from the MSC-Exos in context of regenerative medicine. We also discuss their role as diagnostic and therapeutic targets for regenerative medicine and tissue engineering.

## Overview of MSC-Exo-ncRNA

In the past two decades, advancements in high throughput sequencing technologies and computational approaches have successfully captured and annotated the transcriptome revealing the non-coding transcripts and their functionality in the human genome (67, 68). The extensive information from these in-depth analyses demonstrates the genome complexity, with <2% of the genome encoding than a large percentage (98%) being transcribed into heterogeneous ncRNAs transcripts (69).

**Figure 2:**
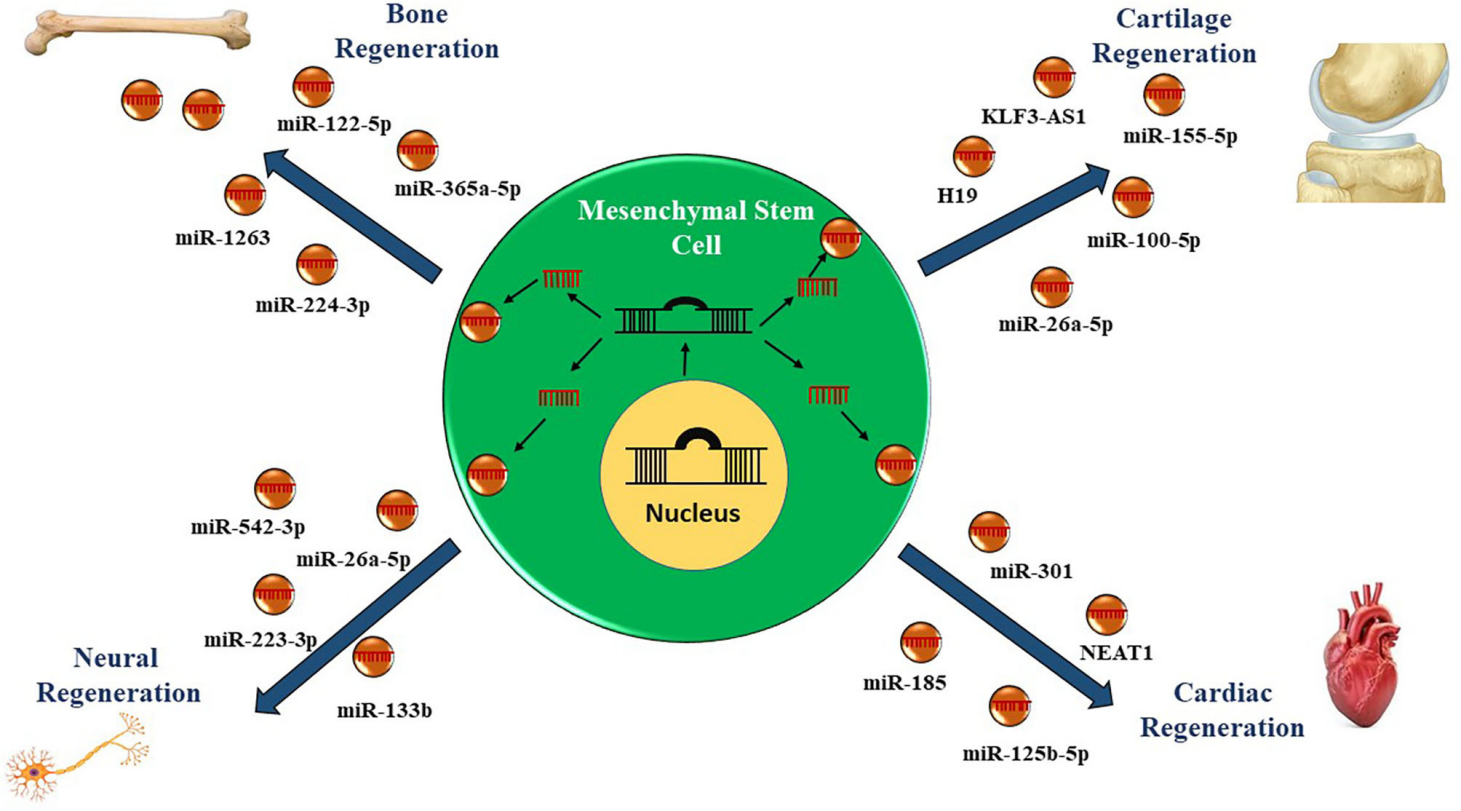
Role of non-coding RNAs in mesenchymal stem cell-derived exosomes in regenerative therapy. Currently, therapies utilizing mesenchymal stem cells derived exosomes enriched in microRNAs and long non-coding RNAs have been employed to regenerate the damaged cells or tissues.

The ncRNAs comprises of a diversified repertoire of endogenous RNA transcripts including short and long (<200 nucleotides) ncRNAs with no protein-coding potential (70, 71). Initially, the ncRNAs were considered as transcriptional junk having no biological function; however, in recent years, numerous studies have depicted the regulatory function of ncRNAs modulating the expression of genes involved in critical biological processes (72). Moreover, the dysregulated expression signatures of ncRNAs are found to be contributing toward the pathogenesis of various diseases by diverse mechanisms (Figure 2) (73–77).

The MSCs are active sources of exosomes with enriched cargo, including lipids, DNA, RNAs (mRNAs, miRNAs, lncRNAs, circRNAs, and piRNAs) proteins. MSC-Exos are also being explored as viable biomarker in diseases. Their cargo of non-coding RNA (ncRNA), including lncRNA and miRNA, is being studied for their role as potential therapeutic strategies in regenerative medicine (Table 1).

**Table 1:** Mesenchymal stem cell-derived exosomes non-coding RNAs in bone regeneration application.

**S. no**.	**Damage/injury**	**Tissue source**	**Exosome ncRNAs**	**Key research finding**	**References**
1.	ONFH	BMSCs	miR-122-5p	Improved ONFH in osteoblasts by downregulating SPRY2 *via* RTK/RAS/MAPK pathway	(78)
2.	ONFH	BMSCs	miR-224-3p	Lower level of mir-224-3p enhanced angiogenesis in endothelial cells by upregulating FIP200	(79)
3.	GIONFH	HUMSCs	miR-365a-5p	Upregulated miR-365a-5p enhanced osteogenesis and prevented GIONFH through activation of Hippo signaling pathways in rats	(80)
4.	DOP	HUMSCs	miR-1263	HUMSCs derived exosome mir-1263 reduces apoptosis of BMSCs in rat DOP *via* miR-1263/Mob1/Hippo signaling pathway	(81)
5.	PMO	BMSCs	miR-186	Exosome miR-186 derived from BMSCs promoted osteogenesis *via* activation of Mob1/Hippo signaling pathway in the ovariectomized (OVX) rat models of PMO	(82)
6.	BF	BMSCs	miR-128-3p	Improved bone healing by inhibiting the expression of miR-128-3p enhanced the bone recovery *via* activation of SMAD5	(83)
7.	BF	BMSCs	miR-126	Hypoxia elicited BMSC-EVs improved bone healing through the enrichment of miR-126	(84)
8.	OA	hMSCs	KLF3-AS1	Promote cartilage repair by sponging miR-206, increasing the expression GIT-1, further attenuating IL1-β induced apoptosis in chondrocytes	(85)
9.	OA	IPFP-MSC	miR-100-5p	Increased cartilage regeneration *via* application of infrapatellar fat pad MSCs derived exosomes overexpressing miR-100-5p	(86)
10.	OA	SMCs-MSC	miR-140-5p	Administration of human synovial muscle cells mesenchymal stem cell (SMSCs) exosome overexpressing miR-140-5p increases proliferation and migration of chondrocytes *via* Wnt signaling and activation of YAP pathway	(87)
11.	OA	UMSCs	H19	Enhanced chondrocyte migration, matrix secretion, apoptosis suppression, senescence suppression, through sponging chondrocytes miR-29b-3p, and upregulating FOXO3 promoting cartilage repair	(88)
12.	OA	hSMSCs	miR-129-5p	Improved cartilage regeneration mitigates IL1-β induced inflammation and apoptosis in chondrocytes *via* suppression of HMGB1 in chondrocytes	(89)
13.	OA	hBMSCs	miR-26a-5p	Enhanced expression of miR-26a-5p alleviates OA pathogenesis *via* downregulation of PTGS2 in synovial fibroblasts	(90)
14.	MI	BMSCs	miR-301	Improved cardiac function in post MI rat models mediated by BMSCs-exosomes expressing miR-301	(91)
15.	MI	BMSCs	miR-185	Administration of miR-185 improved cardiac function, decreased infarct area *via* regulating the SOCS2/JAK/STAT signaling pathways	(82)
16.	I/R	BMSCs	miR-125b	Administration of exosomes expressing miR-125b decreased inflammation, apoptosis of myocardial cells by downregulating SIRT7 in myocardial cells	(92)
17.	I/R	BMSCs	miR-125b-5p	Hypoxia elicited BMSCs exosomes enriched miR-125b-5p enhanced cardiac function and infract size in rat model of MI	(93)
18.	MI	BMSCs	NEAT1	The administration of pre-conditioned MSCs derived exosomes having elevated expression of NEAT lncRNA have cardioprotective effects on MIF-pretreated exosomes to H_2_O_2_-treated cardiomyocytes *via* modulation of the NEAT1/miR-142-3p/FOXO1 axis	(94)
19.	MCAO/R	MSCs	miR-133b	Administration of miR-133b enriched MSCs exosomes increased neurite remodeling in the ischemic boundary zone (IBZ)	(95)
20.	MCAO/R	MSCs	miR-542-3p	Application of miR542-3p enriched exosomes to MCAO/R mice reduced the infarct volume, edema, infiltration of inflammatory cells *via* decreasing the expression of TLR4 activity	(96)
21.	MCAO/R	MSCs	miR-223-3p	The *in vivo* delivery of ex miR-223-3p decreased production of proinflammatory cytokines (IL-1β and IL-6) while increasing the production of the anti-inflammatory cytokine IL-10 and reduced infarct volume mitigating cerebral injury	(97)

*ONFH, Femoral head's osteonecrosis; GIONFH, Glucocorticoid (GC)-induced osteonecrosis of the femoral head; DOP, Disuse osteoporosis; PMO, Post-menopausal osteoporosis; BF, Bone fracture; MAPK, Mitogen Activated Protein Kinase; FIP, Family interacting protein; Mob1, Mob Kinase Activator 1; SMAD5, Mothers against decapentaplegic homolog 5; KLF3-AS1, KLF3 antisense RNA-1; IPFP-MSC, Infrapatellar fat pad-derived mesenchymal stem cell; GIT1, G-protein-coupled receptor kinase interacting protein-1; YAP, Yes-associated protein; FOXO3, Forkhead box O3; HMGB1, High Mobility Group Box 1; PTGS2, Prostaglandin-endoperoxide synthase 2; MI, Myocardial Infraction; IR, Ischemia Reperfusion; SOCS2, Suppressor Of Cytokine Signaling 2; JAK, Janus Kinase 1; STAT, signal transducer and activator of transcription; SIRT7, NAD-dependent deacetylase Sirtuin 7; MCAO/R, Middle cerebral artery occlusion reperfusion*.

## Role of MSC-Exo-ncRNA in Regenerative Medicine

### Bone Regeneration

The regeneration of bone is a dynamic and complicated process that requires the coordination of numerous cell types and biological events such as ossification, osteoinduction, and osteogenesis. In addition to the above-mentioned biological events, series of signaling pathways associated with angiogenesis, proliferation, migration, remodeling, inflammation, and necrosis further play a critical role at the site of bone injury. Bone's inability to regenerate successfully results from the failure in the orchestration of the above-mentioned biological processes and signaling pathways. Bone regeneration failure is one of the significant health problems affecting millions of people worldwide, requiring immediate approaches to improve bone healing. In recent years, studies in regenerative medicine have shown the MSC's potential in promoting bone regeneration through paracrine mechanisms, mainly mediated by exosomes (98–101). The mechanisms governing the beneficial role of MSC-Exos in context to bone regeneration remain, however, elusive.

Current studies have shown that the exosomes from a different source of MSCs mediate ncRNA transport to the injured site to regulate successive biological processes enhancing bone reconstruction. For example, Liao et al. (78) investigated the role of bone marrow MSC (BMSCs)-derived exosomes expressing miR-122-5p on the femoral head's osteonecrosis (ONFH). The authors demonstrated that the expression profile of miR-122-5p was decreased in ONFH, while exosomes carrying miR-122-5p overexpression effectively attenuated ONFH by downregulating SPRY2 *via* the RTK/Ras/mitogen-activated protein kinase (MAPK) signaling pathway (78). Another study suggested that decreased expression of miR-224-3p levels in BMSCs-Exos can attenuate traumatic ONFH by facilitating angiogenesis by upregulating focal adhesion kinase family interacting protein (FIP200) in endothelial cells (79). Similarly, exosomal miR-365a-5p produced by human umbilical cord-derived MSCs (hum-MSCs) was found to alleviate glucocorticoid (GC)-induced osteonecrosis of the femoral head (GIONFH) by promoting osteogenesis *via* activation of the Hippo signaling pathway in rats (80). Moreover, in a hind limb unloading (HLU)-disuse osteoporosis (DOP) rat models, hum-MSC-Exos miR-1263 effectively ameliorated the apoptosis of BMSCs *via* modulating the Mob1/Hippo signaling pathway in the prevention of rat DOP (81).

Interestingly, research efforts have shown that preconditioning can influence the regenerative efficacy of MSC-derived Exos. Using an *in vivo* model of bone fracture, the authors illustrate that hypoxia preconditioning of MSCs enhances exosomal miR-126 by activation of hypoxia-inducible factor 1 α (HIF-1α) and the SPRED1/Ras/Erk signaling pathway. Additionally, knockdown of HIF-1α resulted in decreased expression of MSCs-Exos miR-126, abolishing its protective effect in bone healing (84).

The effects of age on the MSCs-Exos on osteogenic differentiation, osteogenesis, and fracture healing have also been investigated. Bone regeneration is often associated with age, characterized by decreased bone formation due to reduced osteogenic differentiation. Notably, a negative correlation was reported in the expression of miR-128-3p derived from MSCs-Exos and bone regeneration. This *in vitro* experiment further reveals that exosomal miR-128-3p can suppress bone healing by inhibiting the expression of Smad5. Furthermore, reverting the effect of miR-128-3p using antagomir was found to enhance the bone recovery process *via* the increase in Smad5 (83). Additionally, BMSCs exosomal miR-186 promoted osteogenesis *via* MOB Kinase Activator 1A (Mob1)/Hippo signaling pathway in the ovariectomized (OVX) rats model of postmenopausal osteoporosis (PMO) (82).

In conclusion, the above research results substantiate that the beneficial effect of MSC-Exos is mediated by ncRNAs, making them a potential therapeutic candidate in bone regeneration.

### Cartilage Regeneration

In the recent years, the therapeutic roles of exosomes secreted from MSCs have also been assessed regarding cartilage regeneration. Interestingly, the exosomal vesicles derived from MSCs can regulate proliferation, differentiation, and matrix synthesis, indicating this strategy's significance in cartilage regeneration (102, 103). Exosomes' therapeutic mechanisms through ncRNAs in context to cartilage regeneration have, however, only more recently begun to be explored. Recent data suggest that cytokines' inflammatory effect can be regulated by the EVs derived from MSCs containing lncRNA. For instance, the protective effect of exosome lncRNA-KLF3-AS1 derived from hMSCs was investigated in the collagenase-induced rat model of osteoarthritis (OA) and IL-1β-induced OA chondrocytes. The lncRNA-KLF3-AS1 could exert a therapeutic effect by suppressing IL-1β-induced apoptosis in chondrocytes. In addition, *in vivo* investigation further indicated that exosomal KLF3-AS1can promote cartilage repair and chondrocyte proliferation in a rat model of OA (104). These outcomes were mediated by sponging the miR-206 and increasing the expression of G-protein-coupled receptor kinase interacting protein-1 (GIT1) in the chondrocytes (85).

Interestingly, the application of infrapatellar fat pad (IPFP) MSCs-derived exosomes (MSC^IPFP^-Exos) overexpressing miR-100-5p were reported to diminish cartilage deterioration *via* inhibition of the mTOR-autophagy pathway (86). In a rat model of OA, administration of human synovial muscle cells mesenchymal stem cells (SMSCs) exosomes overexpressing miR-140-5p was found to increase the proliferation and migration of chondrocytes *via* Wnt signaling and activation of Yes-associated protein (YAP) in a rat model of OA (87). Similar results were reported when the SMSCs exosomes expressing miR-155-5p showed an anti-apoptotic response in the osteoarthritic chondrocytes and further promoted proliferation and migration, enhanced ECM secretion, and effectively prevented OA in a mouse model (105).

In a separate study, umbilical cord mesenchymal stem cells (U-MSCs) exosomes attenuated osteochondral damage *via* upregulation of lncRNA H19 (88). Further investigation of the role of U-MSCs exosomes demonstrated their ability to transfer the lncRNA H19 to chondrocytes. The exosome-derived lncRNA H19 was notably successful in promoting chondrocyte migration, matrix secretion, apoptosis suppression, as well as senescence suppression, both *in vitro* and *in vivo* by acting as a competing endogenous sponge against miR-29b-3p upregulating FoxO3 (106). To explore the anti-inflammatory effect of exosomes in OA, it was revealed that human synovial mesenchymal stem cells (HS-MSCs) exosomes-derived miR-129-5p could mitigate interleukin-1β (IL-1β)-induced inflammation and apoptosis in chondrocytes *via* suppressing the expression of high mobility group box protein-1 (HMGB1) (89). In another study on a rat model hBMSCs-derived exosome overexpressing miR-26a-5p improved OA pathogenesis *via* downregulating prostaglandin-endoperoxide synthase 2 (PTGS2) expression in synovial fibroblast (90). In another work, the authors recently demonstrated the anti-inflammatory and chondroprotective effect of hBMSCs-derived exosome miR-26a-5p attenuating OA *via* regulation of syndecan-1 (SDC1) (107).

In short, these findings suggest the therapeutic potential of MSCs-Exos ncRNAs in cartilage regeneration.

### Cardiac Regeneration

Mesenchymal stem cells (MSCs) have been studied in great depth regarding their potential use in regenerative medicine. The most notable application is the regeneration of cardiac tissue after myocardial infarct (MI) (108). Cardiomyocyte regenerative capacity is markedly limited as these cells are not thought to enter the cell cycle to undergo division. As a result, injured tissue generally becomes necrotic, and a non-contractile scar tissue forms, consequently limiting the contractile ability to neighbor cells. Due to their limited capacity to regenerate, cardiomyocytes have been the target of recent advents in regenerative medicine as MSCs have been shown to direct cardiac tissue reconstruction following MI (109, 110). Previously, it was suggested that MSCs differentiate into cardiomyocytes and that this process was responsible for repairing cardiac tissue (111, 112). This hypothesis turned out not to be feasible. However, there are some cardiac benefits from treatment with stem cells, including decreased cardiomyocyte apoptosis, reduced fibrosis, enhanced neovascularization and improved left ventricular ejection fraction; due to the exosomes released from the stem cells *in situ*. Because of this effect exosome therapy recapitulates the benefits of stem cell therapy (57), and studies have shown that the activation of cardioprotective pathways obtained by stem cell therapy can be recapitulated by the administration of exosomes obtained from the stem cells (113). Moreover, some studies have demonstrated that exosomes released from cardiac progenitor cells can improve cardiac function in the damaged heart (114, 115).

Recent evidence points to MSC-derived exosomal cargo, including lncRNA and miRNA, which impart these regenerative effects *via* paracrine action on nearby cardiomyocytes (27, 116, 117). Following this observation, exosomes have become desirable therapeutic avenues in regenerative medicine due to their low immunogenicity, low toxicity, stability, and cargo durability.

Li et al. (91) discovered that bone marrow-derived MSC (BM-MSC) exosomes expressing miR-301 protected rat cardiomyocytes from autophagy following MI and improved cardiac function in post-MI rat models. Compared to sham rats, exosome miR-301-treated rats showed higher left ventricular ejection fractions (LVEF) and left ventricular fractional shortening (LVFS) as well as decreased left ventricular end diastolic diameter (LVEDD) and left ventricular end systolic diameter (LVESD) following myocardial infarct. These variables are commonly assessed to measure cardiac function. The LC3-II/LC3-I ratio decreased in exosome miR-301 rat models post myocardial infarct and p62 expression increased, indicating a lower level of autophagy compared to sham models (91).

In another study, administration of BM-MSCs exosomes harboring miR-185 inhibited SOCS2, a member of the SOCS family, which function as repressors of JAK/STAT signaling. SOCS2 is a feedback suppressor of the growth hormone/insulin-like growth factor axis. In mouse models, increased miR-185 decreased the expression of SOCS2 and exosome derived miR-185 delivered to mice post-MI improved cardiac function, decreased collagen I and III, and decreased fibronectin while also decreasing infarct area. Overexpression of SOCS2 reversed the regenerative effects of miR-185. miR-185 decelerated the rate of apoptosis in cardiac tissue post-MI with an observed decrease in BAX expression and increase in Bcl-2 expression (118).

Similar to the effects seen in miR-301 expressing exosomes, Chen et al. observed that exosomes harboring miR-125b increased viability and decreased inflammation and apoptosis of ischemia/reperfusion injury (I/R) rat model myocardial cells by downregulating SIRT7 (92). SIRT7 is in the sirtuin family and functions as a deacetylase to preserve the repressive action of heterochromatin (119). SIRT7 has also been implicated as an apoptosis regulator by hyperacetylating p53 *in vivo* (120).

At the forefront of exosome therapeutic efficacy lie barriers to mimic the exact clinical condition and specific delivery to the affected tissue, limiting the therapeutic efficacy and clinical translation. The pre-conditioning in environments and further conjugating the exosomes to targeted proteins provides another route of establishing desirable qualities exosomes which can be delivered specifically (121, 122).

Recently, Zhu et al. demonstrated a novel mechanism of conjugating CSTSMLKAC peptide [derived from ischemic myocardium-targeted (IMT) peptide], interacting with cardiac troponin 1- to hypoxia-conditioned mouse BM-MSCs (Hypo-exo). The IMT-conjugated exosomes were specifically targeted to myocardial tissue and retained in the ischemic left ventricle vs non-ischemic right ventricle as observed 24 h post-MI. The BM-MSCs (Hypo-exo) were enriched in anti-apoptotic miR-125b-5p. Intramyocardial delivery of Hypo-exo reduced infarct size, improved cardiac function, and significantly decreased apoptosis in mice with permanent-condition MI. Exosome miR-125b-5p treated rats showed increased cardiac function and decreased infarct size compared to control-exosome-treated rats. The pre-conditioning in environments mimicking cardiac ischemia as opposed to exogenously incorporating non-coding RNAs provides another route of establishing desirable qualities in MSC-derived exosomes (93).

Beyond miRNA cargo, lncRNAs such as NEAT1 have been implicated in cardio protection as upregulation of NEAT1 has shown cytoprotective effects in doxorubicin-induced cardiac injury (94). Interestingly one of the studies reported that NEAT1 sponges miR-142-3p, known to regulate FOXO1 expression in an inhibitory manner. FOXO1 belongs to the FOXO family of transcription factors that function to ameliorate oxidative stress and inflammation and increase expression of antioxidant enzymes such as superoxide dismutase (SOD) and catalase in many cell types including cardiac tissue (123).

In another study preconditioning of MSC-derived exosomes with macrophage migration inhibitory factor (MIF), known to be upregulated in cardiac tissue following MI, led to increased levels of NEAT1 lncRNA in pre-conditioned exosomes. Cardioprotective effects were seen following administration of MIF-pretreated exosomes to H_2_O_2_-treated cardiomyocytes *via* modulation of the lncRNA NEAT1/miR-142-3p/FOXO1 axis as seen with decreased levels of caspases 3/7 and 8, decreased reactive oxygen species (ROS) production and lipid peroxidation, and increased SOD expression (124).

Research continues to delve into MSC-derived exosomes' intricacies as both *in vitro* and *in vivo* studies have shown the cytoprotective affects and regenerative capabilities associated with exosome-derived ncRNA cargo. There is much to be learned regarding interactions of exosome-derived ncRNA and host targets. Importantly, discerning the risks associated with delivering anti-apoptotic ncRNA and the potential risk of tumorigenesis of host tissue remains elusive. Targeted exosome delivery to affected organs continues to be explored as this remains a barrier to transitioning exosome-derived therapeutics to clinical settings. It would be interesting to conduct more randomized controlled trials on larger animals other than rats/mice. Taken together all the above findings indicates the role of MSCs-Exos ncRNAs in cardiac regeneration.

## Neurodegenerative Diseases

Neurodegeneration is a persistent barrier to the effective recovery of patients following a cerebrovascular accident (CVA) with ischemic stroke (IS). According to the American Heart Association, IS has become the fifth leading cause of death in the United States (125). A blockage in the cerebral arteries characterizes ischemic stroke, predominantly occurring in the middle cerebral artery (MCA), which subsequently prevents blood flow to the brain. Thrombolytic therapies are the first-line interventions administered following an ischemic stroke, but their effectiveness decreases as the time from stroke to hospital admission increases (126). Lack of oxygen following blockage of the cerebral artery causes downstream cerebral tissue hypoxia and cell death, leading to irreversible damage to brain tissue and accompanying neurocognitive disabilities (127). Researchers have been working to regenerate injured brain tissue following IS using mesenchymal stem cell-derived exosomes as increasing evidence has pointed to the therapeutic role of miRNAs in attenuating cell damage and inflammation following brain injury (128–131).

A primary goal in treating ischemic stroke is decreasing compensatory injury to nerve cells deprived of oxygen downstream of the vascular blockage. Rapid reperfusion is performed to reinstate oxygen to the affected tissue to rescue cells from hypoxia-induced damage, though only a limited time window is allotted for effective reperfusion. Various miRNAs are involved in the mitigation of ischemic stroke, whereby their exogenous introduction to tissues *via* exosomes has been shown to remediate damages associated with CVAs. Xin et al. (95) observed that MCAO/R rats exhibited improved functional outcomes and increased neurite remodeling in the ischemic boundary zone (IBZ) following administration of miR-133b-enriched MSC exosomes.

Inflammatory processes in hypoxic tissue pose another hurdle to effective recovery of patients following IS as persistent activation of inflammatory mediators results in tissue edema and prolonged recruitment of leukocytes to the affected area (132). Cytokines released from injured neural tissue activate inflammatory pathways, of importance being the NF-kB pathway, known for producing proinflammatory cytokines such as IL-1, IL-2, IL-12, and TNF-α, among others. Toll-like receptor 4 (TLR4) is a PRR located on the cell surface of microglia and other macrophages and is involved in intracellular signaling *via* the NF-kB pathway (133). Cai et al. (96) showed that decreasing the expression of TLR4 *via* delivery of miR-542-3p enriched MSC-exosomes to MCAO/R mice reduced infarct volume, edema, and infiltration of inflammatory cells.

Augmenting the inflammatory response through controlling microglial polarization is an attractive route for mitigating cerebral injury following IS. Microglia are macrophages that are key players during the inflammatory process during and after IS and are responsible for both innate and adaptive immunity in the brain. Their polarization into either the M1 or M2 phenotype is characterized by pro-inflammatory or anti-inflammatory cytokine production, respectively (134). Zhao et al. were able to induce M1 to M2 microglial polarization in BV2 mouse microglial cells *via* the introduction of miR-223-3p-enriched MSC-exosomes (ex miR-223-3p). miR-223-3p was found to decrease cysteinyl leukotriene receptor 2 (CysLT2R), which may play a role in the induction of the M1 macrophage phenotype. Treatment with ex miR-223-3p on BV2 cells induced their conversion from the M1 to the M2 phenotype while *in vivo* delivery of ex miR-223-3p decreased production of pro-inflammatory cytokines (IL-1β and IL-6) while increasing the production of the anti-inflammatory cytokine IL-10 and reduced infarct volume in conjunction with improving the functional recovery of rats, including activity, learning, and memory (97).

Neurodegeneration remains an imminent barrier to the recovery of patients following IS. Exosome-derived non-coding RNAs serve many biological processes and are critical mediators in the regeneration and protection of nerve tissue following IS-induced brain injury.

## Cancer Therapeutics

MSC-derived exosomes are currently being explored as viable biomarkers in disease and their cargo of non-coding RNA (ncRNA), including lncRNA and miRNA, are being studied for their role as potent therapeutic strategies in regenerative medicine. This RNA cargo is also being explored in the context of cancer therapeutics while the exosomal skeleton is being looked at as a possible vehicle for delivery of chemotherapeutic agents. The role of MSC-derived exosomes in cancer is quite a contested subject, as numerous reports have provided evidence that various MSC-exosomal lncRNA and miRNA can either inhibit cancer cell proliferation/induce apoptosis or induce metastasis/cancer progression (135–140).

In the recent years, KRAS targeting has emerged to be an efficient therapeutic efficacy in context to lung and pancreatic cancer as KRAS mutations are found in 30% of lung and 90% of pancreatic carcinomas. Yet, KRAS has been denoted an “undruggable” target (in part due to its high affinity for GTP/GDP, smooth surface with limited binding pockets, lack of allosteric binding sites, and extensive post-transcriptional modifications. H3K27me3 methylation is associated with the downregulation of nearby genes, of importance being K-Ras (involved in the Ras/MAPK pathway) (141–143). Xu et al. (135) demonstrated that miR-124 from bone marrow-derived mesenchymal stem cells (BM-MSCs) directly inhibited expression of EZH2, which suppressed proliferation, migration, and epithelial mesenchymal transmission (EMT) while inducing apoptosis in pancreatic cancer cells (Aspc-1 and PANC-1).

Xu and colleagues found that MSC-derived exosomal miR-133b inhibited proliferation, migration, and invasion of U87 glioma cells by decreasing EZH2 expression *in vitro* and inhibited tumor growth *in vivo via* modulating the Wnt/β-catenin signaling pathway by inhibiting the expression of EZH2 (136). In another study, miR-133b was shown to suppress glioblastoma invasion and cell migration *via* downregulation of metalloproteinase 14 (MMP14) in U87 and U251 glioma cells, while miR-133b was also shown to impede proliferation and invasion of glioma due to Sirt1 downregulation (144, 145).

Pakravan et al. found that MSC-derived miR-100 suppressed angiogenesis in breast cancer cells by altering the mTOR/HIF-1α/VEGF signaling axis. mTOR is a protein kinase involved in the PI3K/AKT pathway and is known to drive HIF-1α accumulation, which acts as a transcription factor by binding to promoter regions in genes known as hypoxia-response elements (HREs). HIF-1α binds to the HRE located within the vascular endothelial growth factor (VEGF) promoter to increase expression of VEGF and drive angiogenesis. miR-100 derived from MSCs was shown to decrease expression and protein levels of mTOR, HIF-1α, and VEGF in MDA-MB-231 and MCF-7 breast cancer cell lines (known to overexpress VEGF) when co-cultured with MSCs containing miR-100 (146).

An opposing conclusion was made regarding the therapeutic use of MSC-derived exosomal ncRNA and cancer, whereby expression of various lncRNAs (AGAP2-AS1 and HCP5) promoted stemness and drug resistance in breast cancer and gastric cancer, respectively. Han et al. (140) found that AGAP2-AS1 lncRNA was upregulated in trastuzumab-resistant breast cancer cells co-cultured with MSCs harboring lncRNA AGAP2-AS1. AGAP2-AS1 was shown to interact directly with HuR (known to stabilize CPT1 mRNA) and increase fatty acid oxidation (FAO) to promote stemness and drug resistance. Wu et al. found that MSC-derived HCP5 lncRNA increased FAO in gastric cancer cell lines *via* sponging to miR-3619-5p (found to inhibit expression of CPT1) by affecting the AMPK pathway *via* PPARGC1A and PGC1α, leading to stemness and chemo-resistance. Overexpression of miR-3619-5p significantly decreased expression of PPARGC1A, which decreased PGC1α (product of AMPK pathway), a transcriptional co-activator of FAO genes (137).

The regulation of cancer progression and inhibition *via* modulation of oncogenic pathways using MCS-derived ncRNA is an area that must be studied in greater depth. A solid understanding of ncRNA targets is needed to come to a consensus about the potential use, safety, and benefits of using exosome-derived ncRNAs in cancer therapy.

## Other Diseases

In the retina, the exosomes derived from human U-MSCs (huc-MSCs) expressing miR-126 demonstrated remarkable therapeutic effects by decreasing hyperglycemia associated with retinal inflammation by downregulating the high-mobility group box 1 expression (HMGB1) in diabetic rats and human retinal endothelial cells (HREC) (147). In a murine subretinal fibrosis model, huc-MSCs exosomes containing miR-27b-3p could mitigate subretinal fibrosis by reversing the process of epithelial-mesenchymal transition (EMT) induced by TGF-β2 *via* inhibiting homeobox protein Hox-C6 (HOXC6) (148). The exosomal lncRNA H19 contributed to wound healing by preventing the apoptosis and inflammation of fibroblasts by impairing miR-152-3p-mediated PTEN inhibition in diabetes and foot ulcer disease (DFU) (149). A study of huc-MSCs in an experimental rat model of acute lung injury (ALI) revealed that exosomal miR-22-3p suppressed pathological changes, apoptosis, and NF-κB expression in LPS-treated rats (150).

In summary, MSCs improve the regenerative potential in the above-mentioned diseases by transporting the exosomes enriched ncRNAs having anti-apoptotic, and anti-inflammatory effect.

## Potential Challenges and Prospects Associated With MSC-Exo-ncRNA

The MSCs exosomes impart the regenerative effect on the target cells *via* ncRNAs, possibly making them a promising candidate in the tissue engineering field. However, the mechanisms associated with the ncRNAs packaging, uptake, release, and the underlying regulatory mechanisms are still in their infancy.

Many autoimmune diseases are characterized by an activated inflammatory state resulting in infiltration of the autoreactive immune cells damaging the tissues. Interestingly, MSCs-Exos in many autoimmune diseases have successfully induced immune polarization from an inflammatory to an anti-inflammatory state, mitigating the disease pathogenesis. Much of this might be mediated by the ncRNA repertoire in the exosomes. So, first, it's essential to determine the driving mechanism behind packaging the altered ncRNAs into the exosomes under a specific disease state. Secondly, it would be important to decipher the feedback mechanisms by which specific immune cells can control the exosome release as priming the MSCs to release the exosome more efficiently and enhance the therapeutic efficacy under disease conditions.

In addition to the challenges mentioned above, the low yield of exosomes/MSCs, their purification, storage, optimized dose to be administered would need further development. Therefore, the clinical application must scale up the production of MSCs-Exos without compromising their purity and stability. The concerns raised above impose significant challenges before bringing MSCs-Exos into clinical practice. Nevertheless, working on few of these domains might improve their regenerative potential.

## Conclusion

The potential of the exosomes to recapitulate the therapeutic properties of the stem cells has paved the path toward cell-free alternative therapy, allowing researchers to use them in a multiple diseases and disorders. The underlying factors and the associated mechanisms contributing to the exosomes' regenerative effects have been an area of interest in the past few years. The recent studies depict that the regenerative effects of the exosomes can be ascribed to their ncRNA repertoire. Moreover, modulating the expression of the ncRNAs in the exosomes has allowed researchers to extensively evaluate the protective effects in tissue repair and regenerative medicine. It will be interesting to investigate how the modification in the intracellular and extracellular conditions of the MSCs itself would change the ncRNA repertoire. In addition to that, it would be important to investigate if these changes could eventually generate exosomes having a homogenous expression of the ncRNAs and could be used safely in the clinics.

## Author Contributions

TP and AD conceived the original idea. TP and MJ collected and prepared the literature, reviewed the literature, and wrote the original manuscript. TP, AD, MJ, and ZB contributed to interpretation of the literature. All authors read, discussed, and revised the initial manuscript and read, approved, and contributed to the final manuscript.

## Funding

This work was supported in part by DST-UKEIRI DST/INT/UK/P-50/2012 to AD and TP and by the grant P01GM066730 from the National Institutes of Health, Bethesda, MD, to ZB and TP. The funding body had no role in the design of the study, collection, analysis, interpretation of data, and in writing the manuscript.

## Conflict of Interest

The authors declare that the research was conducted in the absence of any commercial or financial relationships that could be construed as a potential conflict of interest.

## Publisher's Note

All claims expressed in this article are solely those of the authors and do not necessarily represent those of their affiliated organizations, or those of the publisher, the editors and the reviewers. Any product that may be evaluated in this article, or claim that may be made by its manufacturer, is not guaranteed or endorsed by the publisher.
